# Effect of palbociclib plus letrozole on patient-reported health-related quality of life: extended follow-up of the PALOMA-2 trial

**DOI:** 10.1016/j.esmoop.2025.104497

**Published:** 2025-03-18

**Authors:** N. Harbeck, V. Dieras, K.A. Gelmon, R.S. Finn, M. Martin, P. Neven, S. Kim, J. Ma, E. Gauthier, E. Broughton, J. Doan, H.S. Rugo

**Affiliations:** 1 Breast Center, Department of Obstetrics and Gynecology and CCC Munich, LMU University Hospital, Munich, Germany; 2 Department of Medical Oncology, Centre Eugène Marquis, Rennes, France; 3 Department of Medical Oncology, British Columbia Cancer, Vancouver, British Columbia, Canada; 4 Division of Hematology/Oncology, University of California, Los Angeles, USA; 5 Medical Oncology Service, Instituto de Investigacion Sanitaria Gregorio Maranon GEICAM, Universidad Complutense, Madrid, Spain; 6 Department of Oncology, Universitair Ziekenhuis Leuven–Campus Gasthuisberg, Leuven, Belgium; 7 Pfizer Inc, San Diego, USA; 8 Pfizer Inc, Groton, USA; 9 Pfizer Inc, San Francisco, USA; 10 Pfizer Inc, New York, USA; 11 University of California San Francisco Helen Diller Family Comprehensive Cancer Center, San Francisco, USA

**Keywords:** advanced breast cancer, EQ-5D, FACT-B, palbociclib, PALOMA-2, quality of life

## Abstract

**Background:**

Metastatic breast cancer (mBC) remains incurable, highlighting the importance of patient-reported outcomes (PROs) in treatment decision making. In the randomized phase III PALOMA-2 trial, health-related quality of life (HRQoL) was maintained in patients receiving first-line palbociclib plus letrozole compared with placebo plus letrozole after a median follow-up of 22.3 months. However, little is known about HRQoL for patients taking palbociclib for an extended period of time. Here, we report the PRO results from the PALOMA-2 trial after a median follow-up time of 90 months.

**Patients and methods:**

Women with estrogen receptor-positive/human epidermal growth factor receptor 2-negative (ER+/HER2−) mBC were randomly assigned 2 : 1 to receive palbociclib plus letrozole (*n* = 444) or placebo plus letrozole (*n* = 222). HRQoL was assessed with the Functional Assessment of Cancer Therapy-Breast (FACT-B) and EuroQoL five-dimensions three-level (EQ-5D-3L) questionnaires, administered on site on day 1 of cycles 1, 2, and 3 and then every other cycle from cycle 5 until study end. Treatment arm comparisons were made for change from baseline in QoL and time to deterioration in FACT-B (definitive definition, TTDD).

**Results:**

After a median follow-up of 90 months, no significant differences between treatments were observed for overall change from baseline in FACT-B total, FACT-B subscales, and EQ-5D-3L scores. While TTDD did not differ between treatment arms, TTDD was shorter for patients with disease progression versus those without disease progression (hazard ratio 0.644, *P* < 0.001). Individual items assessing side-effects and hair loss favored the palbociclib plus letrozole arm versus the letrozole arm; no treatment difference was observed for items assessing pain.

**Conclusions:**

This extended follow-up analysis of PROs in PALOMA-2 shows continued QoL maintenance for patients with ER+/HER2− mBC receiving long-term palbociclib plus letrozole treatment.

## Highlights

• Considering health-related QoL is an essential component of the metastatic breast cancer treatment decision-making process.
• QoL was assessed by the validated FACT-B and EQ-5D-3L questionnaires.
• This study features the longest follow-up duration for CDK4/6 inhibitor-treatment-based patient-reported outcome data.
• With a 90-month median follow-up, QoL was preserved for patients taking palbociclib + letrozole versus placebo + letrozole.
• Long-term QoL maintenance with a PFS benefit demonstrates that palbociclib is an effective treatment for ER+/HER2− mBC.

## Introduction

Estrogen receptor-positive/human epidermal growth factor receptor 2-negative (ER+/HER2−) metastatic breast cancer (mBC) continues to be a major health concern for people in the USA and globally, with poor survival outcomes for disease that has spread beyond the breast and lymph nodes [5-year survival in the USA, 34%^1^; median overall survival (OS), 37.2-43.4 months in Europe].^2^^,^^3^ Palbociclib, the first-in-class cyclin-dependent kinase 4/6 (CDK4/6) inhibitor,^4^ is known to extend progression-free survival (PFS) in patients with ER+/HER2− mBC when combined with endocrine therapy (ET) in clinical trials,^5^^,^^6^ and this combination is associated with real-world OS benefits.^7^ In the phase III PALOMA-2 trial, PFS was significantly improved with palbociclib plus letrozole compared with placebo plus letrozole [27.6 months versus 14.5 months, hazard ratio (HR) 0.563, 95% confidence interval (CI) 0.461-0.687, *P* < 0.0001] in patients with ER+/HER2− advanced breast cancer (ABC)^5^^,^^6^; however, no significant difference between treatment arms was observed in OS (a secondary endpoint of the trial).^8^ In a large comparative real-world study, however, median OS was significantly longer with palbociclib plus an aromatase inhibitor (AI; *n* = 1324) compared with an AI alone (*n* = 1564, HR 0.76, 95% CI 0.65-0.87, *P* < 0.0001).^7^ Accumulating real-world evidence suggests that patients with mBC receiving palbociclib plus letrozole can have extended survival; in the PARSIFAL-LONG study (*n* = 192), median OS was 61.9 months (95% CI 55.7-71.3 months)^9^ and in an extended follow-up of a phase II Japanese trial (*n* = 42), median OS was 85.4 months (95% CI 64.3 months to not estimable).^10^

Because current therapies for mBC provide survival benefits but are not considered curative, understanding their impact on health-related quality of life (HRQoL) is essential to informed care decisions. Patient-reported outcomes (PROs) are crucial tools for gaining insight into patients’ subjective perception of their care and are now recognized as integral components of clinical trials assessing the safety and efficacy of mBC therapies.^11^^,^^12^ A previous report describing PROs from the PALOMA-2 trial showed that QoL in the first line did not differ from the general population and was maintained over time, after a median follow-up time of 22.3 months.^13^ Notably, improvements in pain scores were reported for the palbociclib plus letrozole arm in PALOMA-2 and the palbociclib plus fulvestrant arm in PALOMA-3.^8^^,^^13^^,^^14^ Here, we report an extended follow-up analysis of PROs for the PALOMA-2 trial after a median follow-up of 90 months.

## Methods

### Study design, participants, and treatments

The PALOMA-2 trial (clinical trial number NCT01740427) was an international, multicenter, double-blind, placebo-controlled, phase III study that enrolled postmenopausal women with ER+/HER2− ABC who had not previously received systemic treatment for their advanced disease.^5^ Adult women (≥18 years of age) were randomly assigned 2 : 1 to receive either palbociclib plus letrozole or placebo plus letrozole (Supplementary Figure S1, available at https://doi.org/10.1016/j.esmoop.2025.104497). Detailed methods have been previously published.^5^^,^^6^^,^^13^ Other inclusion criteria included adequate organ function, an Eastern Cooperative Oncology Group performance status (ECOG PS) of 0-2, and measurable disease according to RECIST version 1.1 or bone-only disease. Exclusion criteria included advanced, symptomatic visceral spread, with the risk of life-threatening complications in the short term. Patients were enrolled between February 2013 and July 2014.^5^ PRO results with an initial data cut-off date of February 2016 were previously reported^5^^,^^13^; this final PRO analysis is based on a data cut-off date of November 2021.

Patients received palbociclib (125 mg/day) or matched placebo orally with food for 21 days of every 28-day cycle. Patients received letrozole (2.5 mg/day) orally on a continuous dosing schedule. Dose reductions, interruptions, and cycle delays were permitted to manage treatment-related toxicity. The study was carried out in accordance with the principles outlined in Good Clinical Practice and the Declaration of Helsinki; local review boards at each investigational center reviewed and approved the study protocol. All participants included in the study provided informed consent.

### Endpoints and assessments

Patient-reported HRQoL assessment was a secondary objective of PALOMA-2. HRQoL was assessed using the Functional Assessment of Cancer Therapy-Breast (FACT-B) and EuroQOL five-dimension three-level (EQ-5D-3L) questionnaires, which are fully validated QoL instruments.^15^^,^^16^ The primary PRO endpoint was the FACT-B total score, while additional endpoints included the Functional Assessment of Cancer Therapy-General (FACT-G) total score, FACT-G subscales, FACT-B trial outcome index (TOI), breast cancer subscale (BCS), EQ-5D index score, and EQ-5D visual analog scale (VAS) general health status score. HRQoL questionnaires were administered on site on day 1 of cycles 1 (baseline assessment), 2, and 3; then every other cycle from cycle 5; and then at the end of study treatment.^13^

The FACT-B (version 4, 1997) is composed of 37-items, which includes the 27-item FACT-G survey and a 10-item BCS survey.^15^^,^^17^ The FACT-G survey is composed of four subscales: physical well-being (PWB), social/family well-being (SWB), emotional well-being (EWB), and functional well-being (FWB). The FACT-B TOI is composed of the PWB + FWB + BCS. Patients rated their responses on a Likert scale where 0 = not at all, 1 = a little bit, 2 = somewhat, 3 = quite a bit, and 4 = very much. Some items were reverse-scored such that a higher total FACT-B score indicated better QoL. Minimum clinically important differences (MCIDs) were defined as seven points for the FACT-B total score, five for the FACT-G total and TOI scores, and two for the BCS score.^18^

The EQ-5D-3L questionnaire is composed of a five-item measure of health status in discrete domains and a VAS that assesses overall health status.^16^^,^^19^ Each health item is scored from 1 to 3, where 1 = no problems, 2 = some problems, and 3 = extreme problems. The EQ-5D VAS is scored on a single scale where 100 = best imaginable health and 0 = worst imaginable health. The MCID was set at 0.06 for the EQ-5D index score and 7 for the EQ-5D VAS score.^20^

Except for the change from baseline (FACT-B, EQ-5D, and EQ-5D VAS) comparisons between treatment arms, all analyses in this extended study were *post hoc*. Between-treatment comparisons of overall change from baseline in FACT-B total score were assessed by demographic and disease characteristic subgroups; subgroups assessed included the presence of visceral metastasis, prior ET, bone-only disease, measurable disease, prior chemotherapy, treatment-free interval (TFI; ≤12 months compared with >12 months), number of disease sites (1, 2, ≥3), and ECOG PS (0, 1/2). Additionally, the time to definitive deterioration (TTDD) on the FACT-B total score was assessed and analyzed by disease progression status. TTDD was defined as a decrease of seven or more points in FACT-B total score without a subsequent observation of a less than seven-point decrease. Finally, between-treatment comparisons of overall change from baseline were made on seven individual items in the FACT-B questionnaire that patients have identified as having particular relevance to mBC, including I have a lack of energy (GP1); I have nausea (GP2); I have pain (GP4); I am bothered by side-effects of treatment (GP5); I am content with the quality of my life right now (GF7); I am bothered by hair loss (B5); and I have certain parts of my body where I experience pain (P2).^21^

### Statistical analyses

The PRO analyses population included patients from the intent-to-treat (ITT) population with a baseline PRO and at least one post-baseline assessment before the end of the study treatment. Completion rates for each PRO instrument were summarized by cycle. For determining change in QoL over time, FACT-B total and subscale scores at each cycle were subtracted from baseline. The primary, prespecified analysis for comparing the two treatment groups was based on a longitudinal, mixed-effects, random-intercept, random-slope model.^22^ Variables used in the model were treatment, time (cycle), and treatment by time, with baseline as a covariate. Time was used as a continuous variable in fitting the mixed-effects model. A restricted maximum likelihood approach was used, and an unstructured covariance matrix was assumed. Similar analyses were conducted for EQ-5D index scores and EQ-5D VAS scores for between-arm comparisons. No adjustments for multiple comparisons were made. TTDD in FACT-B total score was analyzed using an unstratified and stratified (by site of disease, visceral versus nonvisceral) Cox proportional hazards model and log-rank test. Alpha was set at 0.025 for the one-sided log-rank test of TTDD. Between-treatment comparison of overall change from baseline on the FACT-B total score for subgroups and the FACT-B individual items that have particular relevance to mBC were assessed by repeated-measures mixed-effects analyses with baseline as a covariate. All analyses were carried out using SAS v9.2 or higher (SAS Institute; Cary, NC). All *P* values are two-sided unless otherwise stated.

## Results

### Patient and disease characteristics

The PALOMA-2 trial randomly assigned 666 patients (ITT population) to the palbociclib plus letrozole arm (*n* = 444) or placebo plus letrozole arm (*n* = 222). The PRO analysis population included 436 patients from the palbociclib plus letrozole arm and 218 from the placebo plus letrozole arm. Baseline demographics and disease characteristics of the overall population are shown in Supplementary Table S1, available at https://doi.org/10.1016/j.esmoop.2025.104497.

Median patient age was 62 years in the palbociclib plus letrozole arm and 61 years in the placebo plus letrozole arm, with more than one-third of patients aged ≥65 years in both arms. In both arms, most patients were white (77.5%) and had an ECOG PS of 0 or 1 (≥98.0%); more than one-third of patients had *de novo* metastatic disease, and more than two-thirds of patients had two or more disease sites. Baseline patient characteristics in the two treatment arms were well balanced in the ITT poplulation.^5^ More palbociclib dose reductions (*n* = 160, 36.0%), interruptions (*n* = 297, 66.9%), and delays (*n* = 303, 68.2%) were reported compared with placebo (*n* = 3, 1.4%; *n* = 92, 41.4%; *n* = 60, 27.0%, respectively; Supplementary Table S2, available at https://doi.org/10.1016/j.esmoop.2025.104497). The data cut-off date in the present study was November 2021, corresponding to a median follow-up of 90 months (7.5 years). At the data cut-off date, 43 patients remained on palbociclib plus letrozole and 5 patients remained on placebo plus letrozole (Supplementary Figure S1, available at https://doi.org/10.1016/j.esmoop.2025.104497).

### FACT-B total and subscale scores

The percentage of patients who completed at least one question of the FACT-B survey from baseline to cycle 69 ranged from 92.9% to 100% for the palbociclib plus letrozole arm and from 90.9% to 100% for the placebo plus letrozole arm (Supplementary Table S3, available at https://doi.org/10.1016/j.esmoop.2025.104497). Baseline mean [± standard deviation (SD)] FACT-B total scores were similar between the palbociclib plus letrozole arm (101.5 ± 19.1) and the placebo plus letrozole arm (103.1 ± 18.7; Supplementary Table S4, available at https://doi.org/10.1016/j.esmoop.2025.104497), as previously reported.^13^ Whereas the previous QoL assessment from PALOMA-2 had no QoL data beyond cycle 37 (∼3 years after initiation), this study reports QoL data from 136 (31%) patients in the palbociclib plus letrozole arm and 37 (17%) patients in the placebo plus letrozole arm at cycle 39. Data were collected up to cycle 111, although sample sizes became small after cycle 69 (Supplementary Table S3, available at https://doi.org/10.1016/j.esmoop.2025.104497).

**Figure 1:**
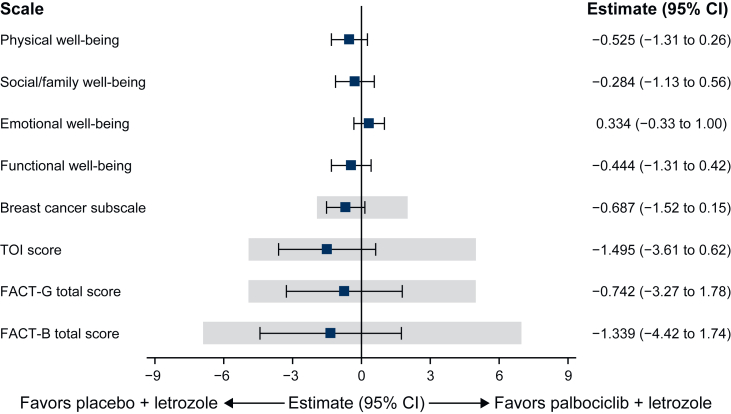
**FACT-B total and subscale scores: between-treatment comparison of overall change from baseline.** Gray boxes indicate minimal clinically important differences for the breast cancer subscale (2 points), TOI index (5 points), FACT-G (5 points) and FACT-B (7 points). CI, confidence interval; FACT-B, Functional Assessment of Cancer Therapy-Breast; FACT-G, Functional Assessment of Cancer Therapy-General; TOI, trial outcome index.

A forest plot shows the comparison between treatment arms in overall change from baseline for FACT-B, FACT-G, and subscales (Figure 1). The mean difference in overall change from baseline in FACT-B total score between the palbociclib plus letrozole arm (−2.36, 95% CI −4.07 to −0.65) and the placebo plus letrozole arm (−1.02, 95% CI −3.58 to 1.54) was modest given the seven-point threshold for clinical importance. The repeated-measures mixed-effects model revealed no significant difference between the two treatment arms (−1.34, 95% CI −4.42 to 1.74; *P* = 0.394; Figure 1), and the interaction between treatment and cycle was also not significant (*P* = 0.214). Mean change from baseline in both treatment arms generally did not reach the FACT-B MCID of −7 points over the course of the study (Supplementary Figure S2, available at https://doi.org/10.1016/j.esmoop.2025.104497). Sample sizes were low for cycles that exceeded the MCID [palbociclib plus letrozole arm: cycle 103 (*n* = 4), cycle 109 (*n* = 1); placebo plus letrozole arm: cycle 53 (*n* = 24), cycle 77 (*n* = 11)].

Baseline mean ± SD FACT-G total scores were similar between treatment arms (palbociclib plus letrozole: 77.7 ± 15.5 versus placebo plus letrozole: 79.0 ± 15.4; Supplementary Table S4, available at https://doi.org/10.1016/j.esmoop.2025.104497). Overall mean change from baseline in FACT-G total score was generally similar between palbociclib plus letrozole (−2.32, 95% CI −3.72 to −0.91) and placebo plus letrozole (−1.57, 95% CI −3.68 to 0.53), with no significant difference between treatment arms (−0.74, 95% CI −3.27 to 1.78, *P* = 0.565; Figure 1). The interaction between treatment and cycle was also not significant (*P* = 0.185). Neither treatment arm reached the FACT-G MCID of −5 points from baseline over the course of the study. There were also no differences in overall change from baseline between the two treatment arms on any of the FACT-G subscales (PWB, SWB, EWB, and FWB; Figure 1). Overall mean changes (95% CI) from baseline for palbociclib plus letrozole versus placebo plus letrozole were: PWB −1.10 (−1.53 to −0.66) versus −0.57 (−1.23 to 0.08); SWB −1.18 (−1.64 to −0.71) versus −0.89 (−1.60 to −0.18); EWB 0.37 (0 to 0.73) versus 0.03 (−0.52 to 0.59); and FWB −0.16 (−0.64 to 0.32) versus 0.28 (−0.44 to 1.01). There were also no significant treatment-by-cycle interactions for any subscale. No significant differences between the palbociclib plus letrozole and placebo plus letrozole arms were noted (Figure 1) for the TOI (−1.36, 95% CI −2.53 to −0.18 versus 0.14, 95% CI −1.63 to 1.90, *P* = 0.166) and the BCS (−0.19, 95% CI −0.65 to 0.28 versus 0.50, 95% CI −0.20 to 1.20, *P* = 0.108). The BCS item assessing pain in body parts was also similar between treatments (−0.14, 95% CI −0.23 to −0.05 versus −0.10, 95% CI −0.24 to 0.04, *P* = 0.647).

### Additional FACT-B analyses

**Figure 2:**
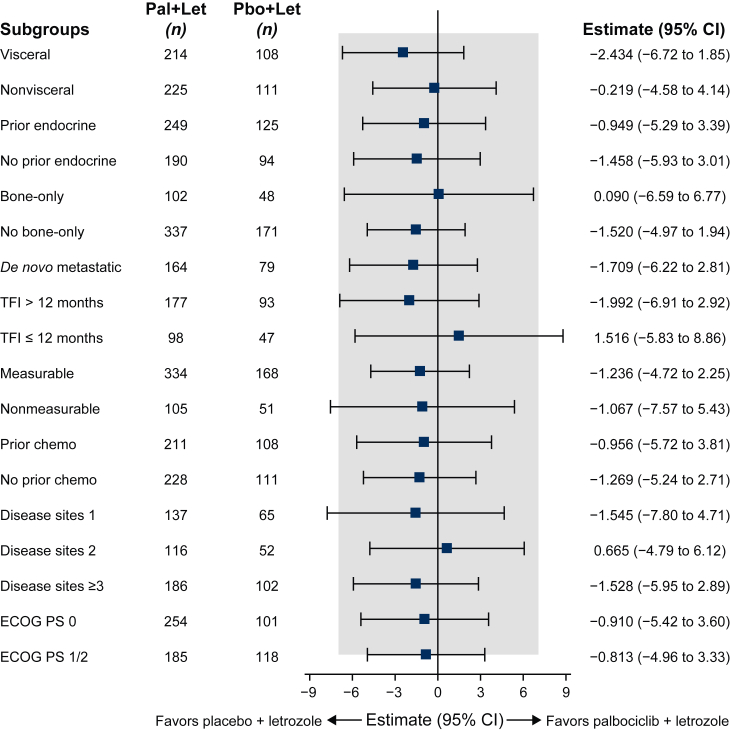
**FACT-B total scores: between-treatment comparison of overall change from baseline by disease characteristics**. FACT-B minimal clinically important difference is 7 points (shaded area). CI, confidence interval; ECOG PS, Eastern Cooperative Oncology Group performance status; Let, letrozole; Pal, palbociclib; Pbo, placebo; TFI, treatment-free interval.

No significant differences between treatment arms were found in the FACT-B total scores when analyzed by subgroups, including the presence or absence of visceral disease, bone-only disease, prior therapy (ET or chemotherapy), or measurable disease. Similarly, no differences were observed for TFI, number of disease sites, or ECOG subgroups. No changes from baseline among the subgroups assessed exceeded the MCID of −7 for FACT-B total scores (Figure 2, Supplementary Table S5, available at https://doi.org/10.1016/j.esmoop.2025.104497).

**Figure 3:**
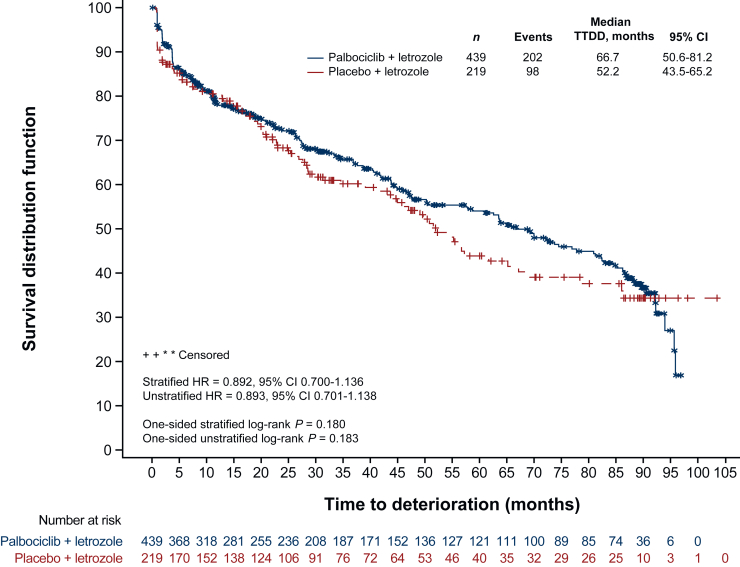
**Kaplan–Meier plot of FACT-B time to deterioration (definitive definition).** Variable used for stratification was site of disease (visceral versus nonvisceral). CI, confidence interval; FACT-B, Functional Assessment of Cancer Therapy-Breast; HR, hazard ratio; TTDD, time to definitive deterioration.

The median TTDD based on FACT-B was 66.7 months (95% CI 50.6-81.2 months) for the palbociclib plus letrozole arm compared with 52.2 months (95% CI 43.5-65.2 months) for the placebo plus letrozole arm (Figure 3). While there was a >14-month numerical advantage in the palbociclib plus letrozole arm over the placebo plus letrozole arm, the difference between treatments was not statistically significant either in the stratified (HR 0.892, 95% CI 0.700-1.136, *P* = 0.180) or unstratified analysis (HR 0.893, 95% CI 0.701-1.138, *P* = 0.183). When analyzed by disease progression status (Supplementary Figure S3, available at https://doi.org/10.1016/j.esmoop.2025.104497), TTDD was significantly longer for patients without disease progression relative to those with disease progression in the palbociclib plus letrozole arm (HR 0.562, 95% CI 0.413-0.764, *P* < 0.001) and for both arms combined (HR 0.644, 95% CI 0.500-0.830, *P* < 0.001); no significant difference in TTDD was observed for the placebo plus letrozole arm (HR 0.864, 95% CI 0.537-1.390, *P* = 0.275), but the sample sizes were relatively small.

Differences in FACT-B oncology-related individual items were also evaluated between treatment groups. For the ‘I am bothered by side-effects of treatment’ GP5 item, patients in the palbociclib plus letrozole arm were significantly less bothered by side-effects during treatment than those in the placebo plus letrozole arm, with a small effect size of 0.278 (*P* < 0.001; Table 1; Supplementary Figure S4A, available at https://doi.org/10.1016/j.esmoop.2025.104497). For the ‘I am bothered by hair loss’ B5 item, patients in the palbociclib plus letrozole arm were significantly less bothered by hair loss during treatment than those in the placebo plus letrozole arm, with a small effect size of 0.413 (*P* < 0.001; Table 1; Supplementary Figure S4B, available at https://doi.org/10.1016/j.esmoop.2025.104497). There were no significant differences between the treatment arms for any of the other individual FACT-B oncology-related items (Table 1).

**Table 1:** FACT-B oncology-related individual items

Item	Baseline SD^a^	Mean difference	Effect size^b^	RM-MEM *P* value
I have a lack of energy (GP1)	1.13	−0.057	0.050	0.428
I have nausea (GP2)	0.80	−0.039	0.049	0.449
I have pain (GP4)	1.15	0.047	0.041	0.543
I am bothered by side-effects of treatment (GP5)	0.96	−0.267	0.278	**0.001**
I am content with the quality of my life right now (GF7)	1.19	−0.043	0.036	0.573
I am bothered by hair loss (B5)	1.34	−0.554	0.413	**<0.001**
I have certain parts of my body where I experience pain (P2)	1.28	−0.039	0.031	0.647

For items GP1, GP2, GP4, GP5, B5, and P2, negative changes indicate improvement from baseline and positive changes indicate deterioration. For item GF7, a positive change indicates improvement from baseline and a negative change indicates deterioration.

*P* values in bold font are statistically significant (<0.05).

FACT-B, Functional Assessment of Cancer Therapy-Breast; RM-MEM, repeated-measures mixed-effects model; SD, standard deviation.

a Baseline SD was calculated from the total population.

b Absolute values of the mean difference were used for computing effect size.

### EQ-5D index and VAS scores

The percentage of patients completing at least one question on the EQ-5D from baseline to cycle 69 ranged from 92.9% to 100% in the palbociclib plus letrozole arm and 87.9% to 100% in the placebo plus letrozole arm. Baseline EQ-5D index and VAS scores were similar between treatment arms (Table 2), as previously reported.^13^ Mean EQ-5D index score while on treatment was similar between the palbociclib plus letrozole arm (0.726, 95% CI 0.71-0.74) and placebo plus letrozole arm (0.719, 95% CI 0.69-0.75). While there was no significant difference between the two treatment arms (0.007, 95% CI −0.03 to 0.04, *P* = 0.700), there was a significant interaction of treatment by cycle (*P* = 0.023). Similar mean on-treatment EQ-5D VAS values were observed between the palbociclib plus letrozole arm (75.8, 95% CI 74.3-77.2) and the placebo plus letrozole arm (76.9, 95% CI 74.7-79.1), with no significant difference between the two arms (−1.15, 95% CI −3.77 to 1.48, *P* = 0.392).

**Table 2:** EQ-5D questionnaire scores at baseline, during treatment, and end of treatment

Health state (at baseline)	Palbociclib + letrozole (*n* = 436)				Placebo + letrozole (*n* = 218)			
**Domains, *n* (%)**	***n***	**No problem**	**Some problem**	**Extreme problem**	***n***	**No problem**	**Some problem**	**Extreme problem**
Mobility	436	268 (61.5)	166 (38.1)	2 (0.5)	215	132 (61.4)	83 (38.6)	0 (0)
Self-care	436	383 (87.8)	50 (11.5)	3 (0.7)	215	189 (87.9)	25 (11.6)	1 (0.5)
Usual activities	436	244 (56.0)	173 (39.7)	19 (4.4)	215	131 (60.9)	78 (36.3)	6 (2.8)
Pain/discomfort	436	135 (31.0)	277 (63.5)	24 (5.5)	215	76 (35.3)	132 (61.4)	7 (3.3)
Anxiety/depression	436	202 (46.3)	212 (48.6)	22 (5.0)	215	99 (46.0)	111 (51.6)	5 (2.3)
**EQ-5D index score**^a^	***n***	**Mean**	**SD**	**95% CI**	***n***	**Mean**	**SD**	**95% CI**
Baseline	436	0.697	0.25	0.67-0.72	215	0.730	0.21	0.70-0.76
Overall on treatment^b^	NA	0.726	NA	0.71-0.74	NA	0.719	NA	0.69-0.75
End of treatment	298	0.651	0.30	0.62-0.69	174	0.674	0.29	0.63-0.72
**EQ-5D VAS**^a^								
Baseline	432	71.3	21.16	69.3-73.3	216	72.3	19.83	69.7-75.0
Overall on treatment^b^	NA	75.8	NA	74.3-77.2	NA	76.9	NA	74.7-79.1
End of treatment	300	71.3	20.64	69.0-73.7	176	72.0	20.25	69.0-75.0

CI, confidence interval; EQ-5D, European quality of life five-dimension; NA, not applicable; SD, standard deviation; VAS, visual analog scale.

a Higher EQ-5D index and VAS scores indicate better health/quality of life.

b Estimated from a repeated-measures mixed-effects model with baseline, treatment, time, and treatment × time interaction terms as covariates.

## Discussion

Patients with mBC are faced with a complex treatment decision-making process influenced by a variety of factors. While survival benefits are a major determinant for evaluating therapies, on-treatment QoL is highly salient to patients.^23^ A key component of QoL identified by patients is periods of time without disease progression, where daily activities can be carried out without interference from disease symptoms or unmanageable side-effects.^24^^,^^25^ Substantial evidence suggests that patients with mBC receive a meaningful PFS benefit when a CDK4/6 inhibitor is added to ET in the first- and later lines of therapy.^5^, ^6^, ^7^^,^^26^, ^27^, ^28^ Therefore, there is a compelling need to demonstrate that these therapies, which provide extended periods of disease stability, also preserve QoL. In this extended analysis of the PALOMA-2 trial, we show that QoL is maintained during treatment with palbociclib plus letrozole compared with placebo plus letrozole, after a median follow-up time of 90 months (>7 years).

Patients initiating treatment in this study had high-level QoL that was comparable to the general population.^17^ Therefore, improvement in QoL was not expected in this first-line setting as would have been the case for patients in later lines, where pain and other symptoms may be more pronounced. Over the course of the study, patients in both treatment arms did not experience reductions in QoL that would be deemed clinically meaningful in the first-line setting. Our results also show that the addition of palbociclib did not significantly impact overall FACT-B outcomes when analyzed by disease characteristics, including subgroups with typically poor outcomes such as patients with visceral disease or with a TFI <12 months.^29^ Although median TTDD on FACT-B scores was not statistically significantly longer with palbociclib plus letrozole compared with placebo plus letrozole, we report a clinically meaningful 14.5-month numerical advantage for palbociclib plus letrozole. Analysis of the Kaplan–Meier curve in Figure 3 shows a clear benefit of palbociclib from month 20 to month 85. At month 90 and beyond, sample sizes become small (*n* ≤ 36 in the palbociclib plus letrozole arm and *n* ≤ 10 in the placebo arm), preventing clear interpretation of the results. Moreover, similar patterns of results were observed with the EQ-5D index and VAS scores, providing further evidence that the addition of palbociclib to letrozole was not detrimental to QoL.

For the overall patient population in our study, TTDD on FACT-B scores was significantly delayed for patients without disease progression compared with those with disease progression, with the effect mostly driven by the palbociclib plus letrozole arm. These findings demonstrate the link between physician-reported progression status and patient-reported QoL. Overall, our results are consistent with the initial PALOMA-2 PRO analysis^13^ and the PALOMA-4 PRO study,^30^ which also show that QoL deteriorates upon disease progression.

Multiple studies have used a variety of PRO instruments to investigate the impact of palbociclib on HRQoL for various patient populations and have reported results ranging from QoL maintenance to improvement. In the PALOMA-3 trial, QoL scores, assessed with the European Organisation for Research and Treatment of Cancer Quality of Life questionnaire (EORTC QLQ-C30) and its breast cancer module (EORTC QLQ-BR23), indicated that patients receiving palbociclib plus fulvestrant had delayed deterioration of QoL and pain symptoms relative to those taking placebo plus fulvestrant.^14^ While results from the FLIPPER trial reported a prolonged time to deterioration in global health status/QoL for placebo plus fulvestrant versus palbociclib plus fulvestrant, QoL assessed by EORTC QLQ-C30 and QLQ-BR23 was maintained in both treatment arms.^31^ In the PEARL trial, patients taking palbociclib plus ET in the metastatic setting were shown to have delayed deterioration in QoL (assessed by EORTC QLQ-30) relative to those receiving chemotherapy.^32^ While a similar median PFS was observed in both arms,^33^ palbociclib was better tolerated than capecitabine, leading to a QoL advantage for patients receiving palbociclib. Studies analyzing subgroups from the PALOMA trials have found that QoL was maintained when palbociclib was added to an AI treatment for Asian patients as well as patients aged ≥65 years.^34^^,^^35^ Overall, our results contribute to the growing body of evidence demonstrating that QoL is preserved with palbociclib treatment for diverse patient populations and irrespective of ET partner.

During treatment with palbociclib plus letrozole in our study, patients reported that they were less bothered by side-effects and hair loss than those treated with placebo plus letrozole. No other differences were observed between the treatment arms when patients were queried about their energy levels, nausea, pain, contentment with their QoL, and pain in certain parts of their bodies. Overall, palbociclib and letrozole are well tolerated, but patients can experience a range of side-effects. Neutropenia is the most common palbociclib side-effect, but febrile neutropenia is rare.^36^ Letrozole can cause pain and fatigue, but severe side-effects are uncommon.^37^ A pooled safety analysis of the PALOMA-1, -2, and -3 trials reported that patients receiving palbociclib plus ET experienced higher incidence of infections, fatigue, and stomatitis than those receiving ET alone, after adjustment for study drug exposure duration.^38^ Notably, the most common hematologic and nonhematologic adverse events tended to occur within the initial months of treatment and tapered off thereafter, suggesting effective adverse event management. Regarding the hair loss item, palbociclib combined with letrozole has been linked to alopecia,^39^ and meta-analyses have shown that the combination of a CDK4/6 inhibitor with an AI increases risk of hair loss.^40^^,^^41^ In the PALOMA-3 trial, patients with endocrine-resistant ER+/HER2− mBC in the placebo plus fulvestrant group was favored on the EORTC QLQ-BR23 ‘upset by hair loss’ item relative to palbociclib plus fulvestrant group, but the sample size was noted as small.^14^ In the previous PALOMA-2 PRO analysis, patients reported significantly greater improvement from baseline on the BCS pain item (P2) when receiving palbociclib plus letrozole than with placebo plus letrozole (change from baseline: −0.256 versus −0.098, *P* = 0.0183).^13^ In this extended analysis with longer follow-up, improvement in pain from baseline changed to −0.142 in the palbociclib plus letrozole group, while it essentially remained unchanged in the placebo plus letrozole group (−0.103), resulting in an overall on-treatment advantage for palbociclib that is no longer significant (*P* = 0.647). Nevertheless, our findings are consistent with a number of randomized controlled trials that have found QoL to be either maintained or improved from baseline among patients treated with palbociclib.^42^ Irrespective of the instruments used to assess pain in these trials, the palbociclib arm exhibited comparable or better QoL than the placebo arm.

This study has certain limitations that should be considered when interpreting the results. If the assumption that missing data were random is incorrect, bias may have been introduced into our analysis. The patients enrolled in PALOMA-2 met specific criteria and may not be representative of the overall population of patients with ER+/HER2− mBC. Despite these limitations, to our knowledge, our study has the longest median follow-up period (90 months) of any PRO-focused data for patients with ER+/HER2− mBC receiving a CDK4/6 inhibitor. HRQoL studies based on similar clinical trials for other CDK4/6 inhibitors have shorter follow-up durations: PRO results from the MONALEESA-2 trial (*n* = 668, 26.4-month median follow-up at interim analysis) showed maintenance of QoL on ribociclib plus letrozole and an early (i.e. ≤15 cycles) pain benefit relative to placebo plus letrozole,^43^^,^^44^ while PRO results from the MONARCH 3 trial (*n* = 493, 26.7-month median follow-up at interim analysis) showed no clinically meaningful differences (except diarrhea) in QoL between patients receiving abemaciclib plus a nonsteroid AI (NSAI) versus placebo plus an NSAI.^45^^,^^46^

This extended analysis of PROs in PALOMA-2 shows that QoL is preserved when palbociclib is added to letrozole for the treatment of ER+/HER2− ABC. The combination of a PFS benefit and long-term high-level QoL maintenance demonstrate that palbociclib is an effective, well-tolerated treatment option for diverse patients with ER+/HER2− mBC. As patients with metastatic disease are living longer, more research is needed to understand the long-term impacts of treatment on the patient journey.
