# Supplementary material for: Effect of palbociclib plus letrozole on patient-reported health-related quality of life: extended follow-up of the PALOMA-2 trial
Source: ESMO Open. 2025 Mar 18;10(4):104497. doi: 10.1016/j.esmoop.2025.104497 (PMC11964640; doi:10.1016/j.esmoop.2025.104497)
Supplement: Supplementary Material [file mmc1.docx]

**Effect of palbociclib plus letrozole on patient-reported health-related quality of life: extended follow-up of the PALOMA-2 trial**

# Authors: Nadia Harbeck^1^, Veronique Dieras^2^, Karen A. Gelmon^3^, Richard S. Finn^4^, Miguel Martin^5^, Patrick Neven^6^, Sindy Kim^7^, Jia Ma^8^, Eric Gauthier^9^, Edward Broughton^10^, Justin Doan^10^, Hope S. Rugo^11^

# Supplemental materials

**Table S1.** Baseline demographic and disease characteristics (ITT population)

| **Demographic or Disease Characteristic** | **Palbociclib + Letrozole**  **(n = 444)** | **Placebo + Letrozole**  **(n = 222)** |
| --- | --- | --- |
| Age |  |  |
| Median, y (range) | 62 (30–89) | 61 (28–88) |
| < 65 y, n (%) | 263 (59.2) | 141 (63.5) |
| ≥ 65 y, n (%) | 181 (40.8) | 81 (36.5) |
| Mean, y (SD) | 61.7 (10.6) | 60.6 (11.2) |
| Race, n (%) |  |  |
| White | 344 (77.5) | 172 (77.5) |
| Non-white | 100 (22.5) | 50 (22.6) |
| Disease site, n (%) |  |  |
| Visceral disease | 214 (48.2) | 110 (49.5) |
| Nonvisceral disease | 230 (51.8) | 112 (50.5) |
| Bone-only | 103 (23.2) | 48 (21.6) |
| Not bone-only | 341 (76.8) | 174 (78.4) |
| Prior adjuvant or neoadjuvant therapies, n (%) |  |  |
| Prior endocrine therapy | 249 (56.1) | 126 (56.8) |
| No prior endocrine therapy | 195 (43.9) | 96 (43.2) |
| Chemotherapy | 213 (48.0) | 109 (49.1) |
| No chemotherapy | 231 (52.0) | 113 (50.9) |
| Measurable disease, n (%) |  |  |
| Yes | 338 (76.1) | 171 (77.0) |
| No | 106 (23.9) | 51 (23.0) |
| ECOG PS, n (%) |  |  |
| 0 | 257 (57.9) | 102 (45.9) |
| 1 | 178 (40.1) | 117 (52.7) |
| 2 | 9 (2.0) | 3 (1.4) |
| Stage of disease at initial diagnosis |  |  |
| I−III | 260 (58.6) | 137 (61.7) |
| IV | 138 (31.1) | 72 (32.4) |
| Unknown, other, or missing | 46 (10.4) | 13 (5.9) |
| Number of involved disease sites |  |  |
| 1 | 138 (31.1) | 66 (29.7) |
| 2 | 117 (26.4) | 52 (23.4) |
| 3 | 112 (25.2) | 61 (27.5) |
| 4 | 52 (11.7) | 29 (13.1) |
| > 4 | 25 (5.6) | 14 (6.3) |
| Disease-free interval (based on CRF) since completion of prior (neo)adjuvant therapy |  |  |
| De novo metastatic | 167 (37.6) | 81 (36.5) |
| ≤ 12 mo from adjuvant to recurrence | 99 (22.3) | 48 (21.6) |
| > 12 mo from adjuvant to recurrence or de novo advanced disease | 178 (40.1) | 93 (41.9) |

CRF, case report form; ECOG PS, Eastern Cooperative Oncology Group performance status; ITT, intent-to-treat; SD, standard deviation.

**Table S2**. Palbociclib, placebo, and letrozole dose reduction, interruption and delay in all cycles combined (ITT population).

|  | **Palbociclib + Letrozole**  **(n = 444)** | **Placebo + Letrozole**  **(n = 222)** |
| --- | --- | --- |
| **Palbociclib or placebo** |  |  |
| Number of patients with at least 1 dose reduction (%)^a^ | 160 (36.0) | 3 (1.4) |
| Total number of patients with dose reduction to 100 mg (%) (1 dose reduction) | 160 (36.0) | 3 (1.4) |
| 100 mg to 75 mg (%) (2 dose reductions) | 63 (14.2) | 0 |
| Patients with dosing interruption (%)^b^ | 297 (66.9) | 92 (41.4) |
| Patients with cycle delay (%)^c^ | 303 (68.2) | 60 (27.0) |
| **Letrozole** |  |  |
| Patients with dosing interruption (%)^b^ | 233 (52.5) | 97 (43.7) |

^a^Dose reduction: Any dose reduction from the initial prescribed dose, regardless of its duration.

^b^Dosing interruption included missed dose collected on the case report form and dose with 0 mg administered.

^c^Cycle delay is defined as any delay of the cycle start beyond 31 days for any given cycle.

ITT, intent-to-treat.

**Table S3.** FACT-B completion rates (ITT population)^a^

|  | **Palbociclib + Letrozole**  **(n = 444)** | | | **Placebo + Letrozole**  **(n = 222)** | | | |  |
| --- | --- | --- | --- | --- | --- | --- | --- | --- |
|  | **n^b^** | **≥ 1 Question** | **No Questions** | | **n^b^** | **≥ 1 Question** | **No Questions** | |
| Baseline | 444 (100) | 441 (99.3) | 3 (0.7) | | 222 (100) | 221 (99.5) | 1 (0.5) | |
| Cycle^c^ 2 | 436 (98.2) | 426 (97.7) | 10 (2.3) | | 218 (98.2) | 214 (98.2) | 4 (1.8) | |
| Cycle 3 | 428 (96.4) | 421 (98.4) | 7 (1.6) | | 207 (93.2) | 205 (99.0) | 2 (1.0) | |
| Cycle 5 | 397 (89.4) | 393 (99.0) | 4 (1.0) | | 175 (78.8) | 172 (98.3) | 3 (1.7) | |
| Cycle 7 | 367 (82.7) | 363 (98.9) | 4 (1.1) | | 158 (71.2) | 153 (96.8) | 5 (3.2) | |
| Cycle 9 | 359 (80.9) | 352 (98.1) | 7 (1.9) | | 149 (67.1) | 146 (98.0) | 3 (2.0) | |
| Cycle 11 | 329 (74.1) | 324 (98.5) | 5 (1.5) | | 136 (61.3) | 134 (98.5) | 2 (1.5) | |
| Cycle 13 | 291 (65.5) | 284 (97.6) | 7 (2.4) | | 124 (55.9) | 120 (96.8) | 4 (3.2) | |
| Cycle 15 | 282 (63.5) | 277 (98.2) | 5 (1.8) | | 116 (52.3) | 113 (97.4) | 3 (2.6) | |
| Cycle 17 | 261 (58.8) | 253 (96.9) | 8 (3.1) | | 102 (45.9) | 100 (98.0) | 2 (2.0) | |
| Cycle 19 | 240 (54.1) | 235 (97.9) | 5 (2.1) | | 91 (41.0) | 89 (97.8) | 2 (2.2) | |
| Cycle 21 | 230 (51.8) | 224 (97.4) | 6 (2.6) | | 85 (38.3) | 84 (98.8) | 1 (1.2) | |
| Cycle 23 | 215 (48.4) | 206 (95.8) | 9 (4.2) | | 79 (35.6) | 77 (97.5) | 2 (2.5) | |
| Cycle 25 | 205 (46.2) | 199 (97.1) | 6 (2.9) | | 70 (31.5) | 70 (100) | 0 | |
| Cycle 27 | 191 (43.0) | 187 (97.9) | 4 (2.1) | | 65 (29.3) | 64 (98.5) | 1 (1.5) | |
| Cycle 29 | 174 (39.2) | 170 (97.7) | 4 (2.3) | | 62 (27.9) | 62 (100) | 0 | |
| Cycle 31 | 167 (37.6) | 162 (97.0) | 5 (3.0) | | 54 (24.3) | 54 (100) | 0 | |
| Cycle 33 | 160 (36.0) | 157 (98.1) | 3 (1.9) | | 52 (23.4) | 51 (98.1) | 1 (1.9) | |
| Cycle 35 | 149 (33.6) | 148 (99.3) | 1 (0.7) | | 44 (19.8) | 42 (95.5) | 2 (4.5) | |
| Cycle 37 | 141 (31.8) | 138 (97.9) | 3 (2.1) | | 40 (18.0) | 40 (100) | 0 | |
| Cycle 39 | 140 (31.5) | 136 (97.1) | 4 (2.9) | | 37 (16.7) | 37 (100) | 0 | |
| Cycle 41 | 125 (28.2) | 119 (95.2) | 6 (4.8) | | 37 (16.7) | 37 (100) | 0 | |
| Cycle 43 | 118 (26.6) | 113 (95.8) | 5 (4.2) | | 33 (14.9) | 30 (90.9) | 3 (9.1) | |
| Cycle 45 | 119 (26.8) | 116 (97.5) | 3 (2.5) | | 30 (13.5) | 30 (100) | 0 | |
| Cycle 47 | 111 (25.0) | 109 (98.2) | 2 (1.8) | | 28 (12.6) | 28 (100) | 0 | |
| Cycle 49 | 105 (23.6) | 103 (98.1) | 2 (1.9) | | 28 (12.6) | 27 (96.4) | 1 (3.6) | |
| Cycle 51 | 98 (22.1) | 95 (96.9) | 3 (3.1) | | 28 (12.6) | 27 (96.4) | 1 (3.6) | |
| Cycle 53 | 95 (21.4) | 94 (98.9) | 1 (1.1) | | 27 (12.2) | 25 (92.6) | 2 (7.4) | |
| Cycle 55 | 87 (19.6) | 87 (100) | 0 | | 22 (9.9) | 20 (90.9) | 2 (9.1) | |
| Cycle 57 | 84 (18.9) | 81 (96.4) | 3 (3.6) | | 21 (9.5) | 21 (100) | 0 | |
| Cycle 59 | 81 (18.2) | 77 (95.1) | 4 (4.9) | | 18 (8.1) | 17 (94.4) | 1 (5.6) | |
| Cycle 61 | 75 (16.9) | 71 (94.7) | 4 (5.3) | | 14 (6.3) | 14 (100) | 0 | |
| Cycle 63 | 76 (17.1) | 72 (94.7) | 4 (5.3) | | 14 (6.3) | 14 (100) | 0 | |
| Cycle 65 | 78 (17.6) | 75 (96.2) | 3 (3.8) | | 13 (5.9) | 13 (100) | 0 | |
| Cycle 67 | 75 (16.9) | 71 (94.7) | 4 (5.3) | | 13 (5.9) | 13 (100) | 0 | |
| Cycle 69 | 70 (15.8) | 65 (92.9) | 5 (7.1) | | 13 (5.9) | 13 (100) | 0 | |
| Cycle 71 | 68 (15.3) | 65 (95.6) | 3 (4.4) | | 12 (5.4) | 12 (100) | 0 | |
| Cycle 73 | 64 (14.4) | 63 (98.4) | 1 (1.6) | | 12 (5.4) | 12 (100) | 0 | |
| Cycle 75 | 64 (14.4) | 60 (93.8) | 4 (6.3) | | 11 (5.0) | 11 (100) | 0 | |
| Cycle 77 | 59 (13.3) | 55 (93.2) | 4 (6.8) | | 11 (5.0) | 11 (100) | 0 | |
| Cycle 79 | 57 (12.8) | 51 (89.5) | 6 (10.5) | | 11 (5.0) | 11 (100) | 0 | |
| Cycle 81 | 57 (12.8) | 54 (94.7) | 3 (5.3) | | 10 (4.5) | 9 (90) | 1 (10) | |
| Cycle 83 | 50 (11.3) | 48 (96.0) | 2 (4.0) | | 8 (3.6) | 8 (100) | 0 | |
| Cycle 85 | 48 (10.8) | 46 (95.8) | 2 (4.2) | | 8 (3.6) | 8 (100) | 0 | |
| Cycle 87 | 47 (10.6) | 45 (95.7) | 2 (4.3) | | 8 (3.6) | 8 (100) | 0 | |
| Cycle 89 | 41 (9.2) | 38 (92.7) | 3 (7.3) | | 8 (3.6) | 8 (100) | 0 | |
| Cycle 91 | 41 (9.2) | 39 (95.1) | 2 (4.9) | | 8 (3.6) | 8 (100) | 0 | |
| Cycle 93 | 36 (8.1) | 33 (91.7) | 3 (8.3) | | 7 (3.2) | 7 (100) | 0 | |
| Cycle 95 | 32 (7.2) | 28 (87.5) | 4 (12.5) | | 6 (2.7) | 6 (100) | 0 | |
| Cycle 97 | 23 (5.2) | 19 (82.6) | 4 (17.4) | | 6 (2.7) | 6 (100) | 0 | |
| Cycle 99 | 15 (3.4) | 13 (86.7) | 2 (13.3) | | 4 (1.8) | 4 (100) | 0 | |
| Cycle 101 | 8 (1.8) | 8 (100) | 0 | | 2 (0.9) | 2 (100) | 0 | |
| Cycle 103 | 4 (0.9) | 4 (100) | 0 | | 1 (0.5) | 1 (100) | 0 | |
| Cycle 105 | - | - | - | | 1 (0.5) | 1 (100) | 0 | |
| Cycle 107 | - | - | - | | 1 (0.5) | 1 (100) | 0 | |
| Cycle 109 | 1 ( 0.2) | 1 ( 100) | 0 | | 1 (0.5) | 1 (100) | 0 | |
| Cycle 111 | - | - | - | | 1 (0.5) | 1 (100) | 0 | |
| EOT | 255 (57.4) | 211 (82.7) | 45 (17.3) | | 146 (65.8) | 129 (88.4) | 17 (11.6) | |

EOT, end of treatment; FACT-B, Functional Assessment of Cancer Therapy–Breast; ITT, intent-to-treat; PRO, patient-reported outcome.

^a^Values are presented as n (%).

^b^Number of patients eligible to complete the questionnaire at the corresponding visit.

Rates of missing PRO data were similar between the 2 treatment arms. The main reasons for missing PROs were patient mentally or physically unable to complete questionnaire, patient refusal to complete questionnaire, or other.

^c^Each cycle was 28 days; cycle 111 = 102.2 months of follow-up.

**Table S4.** Baseline FACT-B scores.

| **Domain** | **Palbociclib + Letrozole**  **(n = 436)**  **Mean (SD)** | **Placebo + Letrozole**  **(n = 218)**  **Mean (SD)** |
| --- | --- | --- |
| Physical well-being | 21.9 (5.5) | 21.8 (5.4) |
| Social/family well-being | 21.8 (5.9) | 22.2 (5.6) |
| Emotional well-being | 16.3 (4.7) | 16.6 (4.7) |
| Functional well-being | 17.5 (6.0) | 18.2 (6.0) |
| Breast cancer subscale | 24.0 (5.6) | 24.2 (5.5) |
| Trial Outcome Index | 63.4 (13.6) | 64.3 (13.2) |
| FACT-G total | 77.7 (15.5) | 79.0 (15.4) |
| FACT-B total | 101.5 (19.1) | 103.1 (18.7) |
| BCS pain item (P2) | 1.8 (1.3) | 1.8 (1.3) |

A higher score indicates better quality of life for FACT-B, FACT-G, and all subscales. A higher score on the FACT-B BCS pain item indicates greater pain severity. BCS, breast cancer subscale; FACT-B, Functional Assessment of Cancer Therapy-Breast; FACT-G, Functional Assessment of Cancer Therapy-General; P2, "I have certain parts of my body where I experience pain”; SD, standard deviation.

**Table S5.** Between treatment comparison of FACT-B total score change from baseline among subgroups.

|  | **Palbociclib + Letrozole** | | | **Placebo + Letrozole** | | | **Palbociclib + Letrozole -**  **Placebo + Letrozole** | | |
| --- | --- | --- | --- | --- | --- | --- | --- | --- | --- |
|  | **n** | **Estimated Mean** | **95% CI** | **n** | **Estimated Mean** | **95% CI** | **Estimated Mean** | **95% CI** | ***P* value** |
| Visceral disease | 241 | -2.026 | -4.41, 0.36 | 108 | 0.408 | -3.16, 3.98 | -2.434 | -6.72, 1.85 | 0.265 |
| Nonvisceral disease | 225 | -2.565 | -4.99, -0.13 | 111 | -2.346 | -5.97, 1.28 | -0.219 | -4.58, 4.14 | 0.922 |
| Prior ET | 249 | -3.369 | -5.69, -1.04 | 125 | -2.420 | -6.09, 1.25 | -0.949 | -5.29, 3.39 | 0.668 |
| No prior ET | 190 | -1.119 | -3.69, 1.45 | 94 | 0.339 | -3.32, 4.00 | -1.458 | -5.93, 3.01 | 0.523 |
| Bone-only disease | 102 | -5.053 | -8.46, -1.65 | 48 | -5.143 | -10.89, 0.60 | 0.090 | -6.59, 6.77 | 0.979 |
| Not bone-only disease | 337 | -1.575 | -3.55, 0.40 | 171 | -0.055 | -2.90, 2.79 | -1.520 | -4.97, 1.94 | 0.389 |
| De novo disease | 164 | -0.065 | -2.64, 2.51 | 79 | 1.645 | -2.07, 5.36 | -1.709 | -6.22, 2.81 | 0.458 |
| TFI > 12 months | 177 | -4.689 | -7.42, -1.96 | 93 | -2.697 | -6.79, 1.39 | -1.992 | -6.91, 2.92 | 0.427 |
| TFI ≤ 12 months | 98 | -1.527 | -5.37, 2.32 | 47 | -3.043 | -9.32, 3.24 | 1.516 | -5.83, 8.86 | 0.686 |
| Measurable disease | 334 | 1.604 | -3.59, 0.38 | 168 | -0.368 | -3.24, 2.51 | -1.236 | -4.72, 2.25 | 0.487 |
| Nonmeasurable disease | 105 | -4.847 | -8.20, -1.49 | 51 | -3.780 | -9.35, 1.79 | -1.067 | -7.57, 5.43 | 0.747 |
| Prior CT | 211 | -3.859 | -6.49, -1.23 | 108 | -2.904 | -6.88, 1.07 | -0.956 | -5.72, 3.81 | 0.694 |
| No prior CT | 228 | -0.936 | -3.16, 1.29 | 111 | 0.333 | -2.97, 3.64 | -1.269 | -5.24, 2.71 | 0.531 |
| 1 disease site | 137 | -6.517 | -9.83, -3.20 | 65 | -4.971 | -10.27, 0.33 | -1.545 | -7.80, 4.71 | 0.628 |
| 2 disease sites | 116 | -1.727 | -4.66, 1.21 | 52 | -2.392 | -7.01, 2.23 | 0.665 | -4.79, 6.12 | 0.811 |
| ≥ 3 disease sites | 186 | 0.361 | -2.23, 2.95 | 102 | 1.889 | -1.70, 5.48 | -1.528 | -5.95, 2.89 | 0.498 |
| ECOG PS 0 | 254 | -4.678 | -7.01, -2.34 | 101 | -3.768 | -7.63, 0.10 | -0.910 | -5.42, 3.60 | 0.693 |
| ECOG PS 1/2 | 185 | 0.866 | -1.62, 3.35 | 118 | 1.679 | -1.64, 4.99 | -0.813 | -4.96, 3.33 | 0.701 |

CI, confidence interval; CT, chemotherapy; ECOG PS, Eastern Cooperative Oncology Group performance status; ET, endocrine therapy; FACT-B, Functional Assessment of Cancer Therapy–Breast; TFI, treatment-free interval.

**Figure S1.** PALOMA-2 CONSORT diagram.


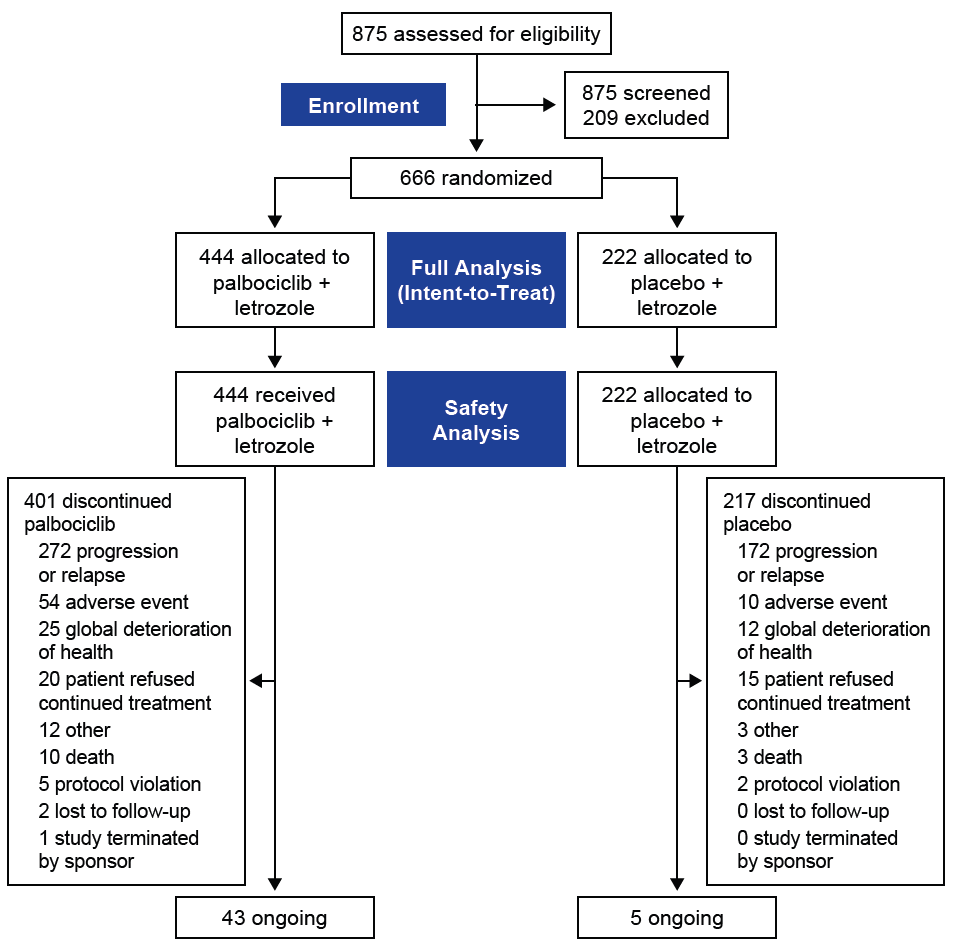


Patient disposition at end of treatment, February 8, 2022.

**Figure S2.** FACT-B total score change from baseline over time in the (A) palbociclib + letrozole arm and (B) placebo + letrozole arm.


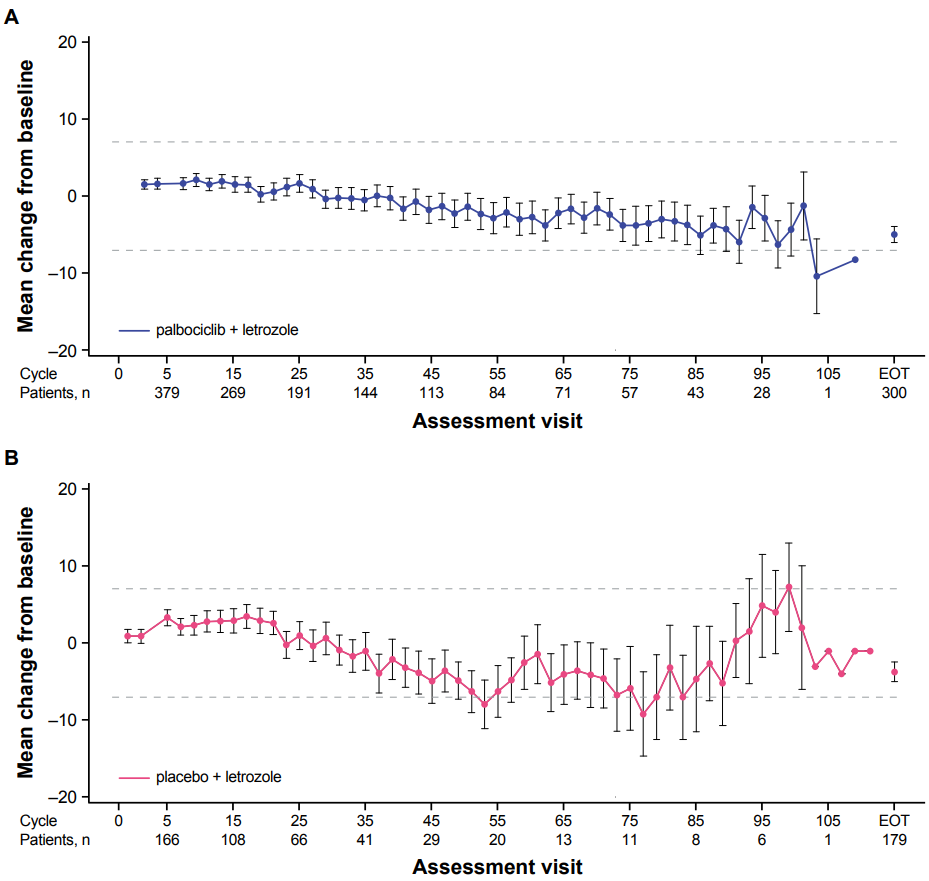


Dashed lines indicate minimal clinically important differences (7 points). Vertical bars indicate standard errors. A positive change indicates quality of life improvement from baseline and a negative change indicates deterioration.

EOT, end of treatment; FACT-B, Functional Assessment of Cancer Therapy-Breast

**Figure S3**. Kaplan–Meier plots of FACT-B time to deterioration (definitive definition) comparing patients with disease progression with those that did not progress in (A) palbociclib + letrozole arm; (B) placebo + letrozole arm; and (C) both arms combined.


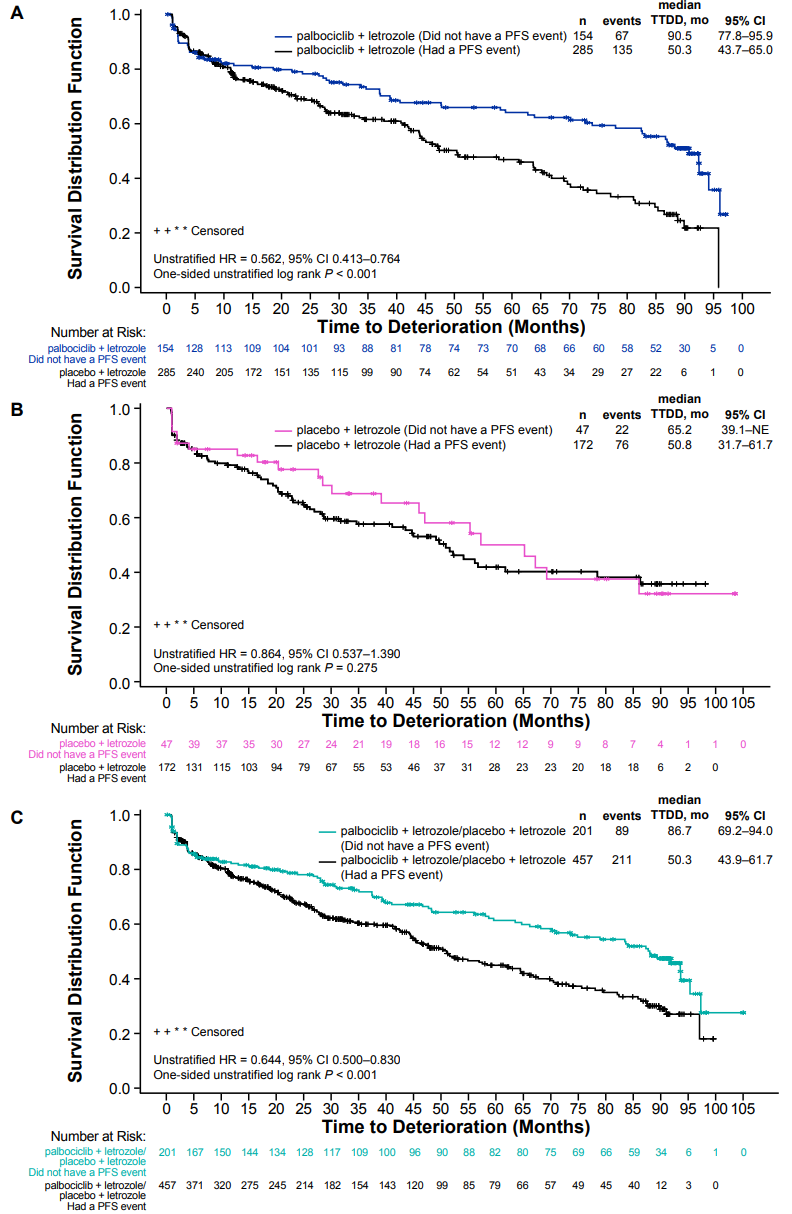


CI, confidence interval; HR, hazard ratio; mo, months; NE, not estimable; PFS, progression-free survival; TTDD, time to definitive deterioration.

**Figure S4.** Comparison between treatment arms by cycle of patients being bothered by (A) side effects of treatment (GP5) and (B) hair loss (B5).


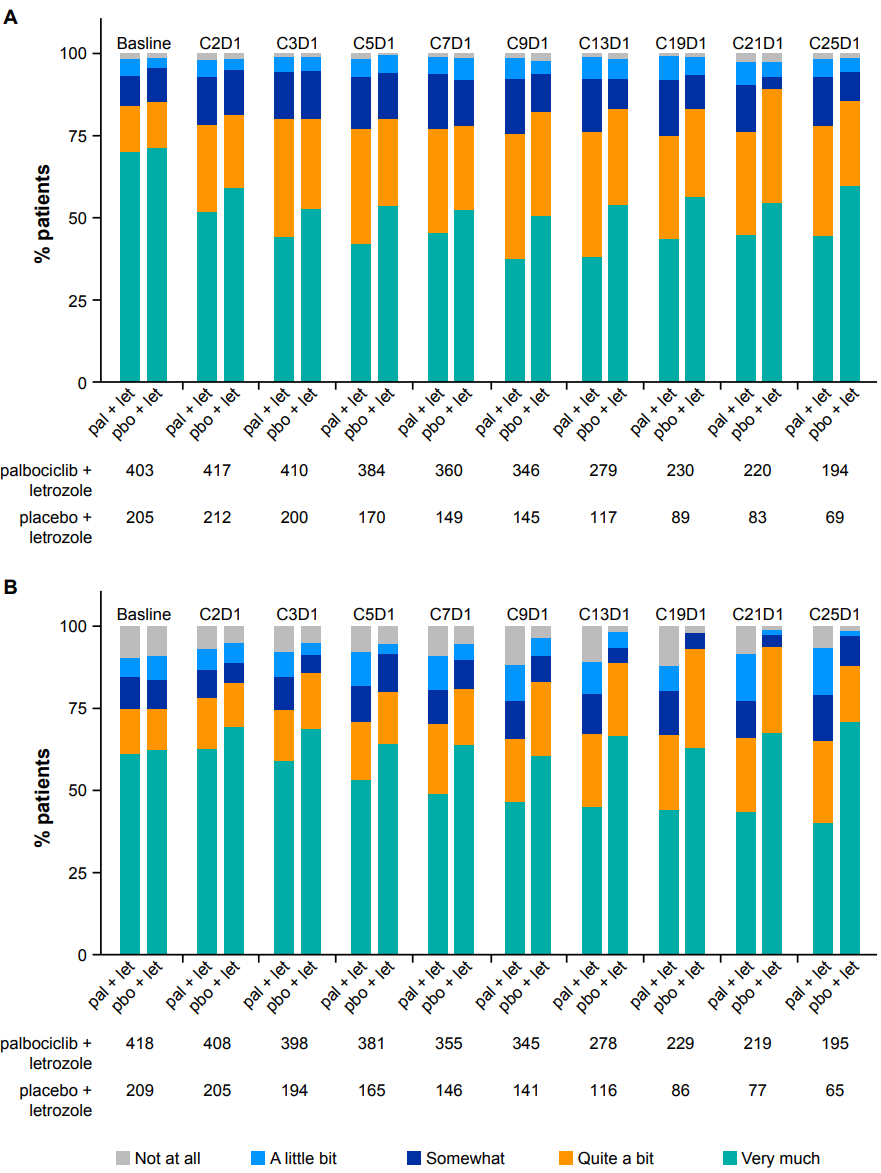


C, cycle; D, day; let, letrozole; pal, palbociclib; pbo, placebo.
